# Influence of intimate partner violence and male involvement on maternal healthcare services utilisation in Nigeria

**DOI:** 10.3389/fgwh.2024.1353117

**Published:** 2024-03-15

**Authors:** O. M. Adetutu, F. F. Oyinlola, T. E. Oyelakin, F. L. Ofili

**Affiliations:** Department of Demography and Social Statistics, Faculty of Social Sciences, Obafemi Awolowo University, Ile-Ife, Nigeria

**Keywords:** intimate partner violence, male involvement, maternal health uptake, gender and power, Nigeria

## Abstract

**Introduction:**

Low maternal health care services utilisation, especially antenatal care attendance and skilled birth attendance, has been documented to be responsible for maternal mortality and morbidity in Nigeria. While available evidence suggests mixed findings on uptake of maternal health care services in the context of abusive spousal relationships, male involvement in household and health decision-making has been established to promote uptake of maternal health care services. Yet, studies which consider mediating influence of intimate partner violence on male involvement and maternal health care services uptake are sorely missing in Nigeria. We hypothesised that maternal health care services uptake in abusive marital unions has implications for male involvement in pregnancy care and this has been largely overlooked in Nigeria.

**Materials and methods:**

This study extracted data from the 2018 Nigeria Demographic and Health Survey (NDHS). The 2018 NDHS is a nationally representative secondary data which collected population, demographic and health information on women, men and households in Nigeria. The secondary data used a two-stage stratified and multistage sampling technique to collect information from the respondents. In this study, data were extracted for women who were sexually active, within the reproductive age (15–49 years) and not pregnant in five years prior the survey (*n* = 7,847).

**Results:**

The results indicated (77%) antenatal care attendance and (47%) skilled delivery. The mediating influence of IPV on male involvement resulted in women who experienced sexual violence more likely to use heath facility for antenatal care (OR = 3.20; C.I: 1.20–8.50). Women whose partners were involved in health decision making had lower odds of antenatal care attendance (OR = 0.64; C.I: 0.44–0.94). Also, women whose partners were involved in spending their earnings had lower probability of antenatal care attendance (OR = 0.72; C.I: 0.55–0.96). Yet, the mediating influence of intimate partner violence on male involvement resulted in a lower likelihood of use of skilled delivery for emotionally abused women (OR = 0.58; C.I: 0.39–0.85). Women whose partners were involved in spending their earnings had higher odds of using skilled delivery (OR = 2.15; C.I: 1.79–2.56). Yet, women whose partners were involved in their health decision-making had lower odds of using skilled delivery (OR = 0.46; C.I: 0.34–0.62).

**Conclusion:**

This study held the philosophical stance that intimate partner violence mediated the influence of male involvement on maternal health care uptake while intimate partner violence had an inconsistent influence on maternal health care uptake. Policies and interventions should aim at addressing deep-rooted gender norms which promote IPV and limit male involvement in pregnancy care in Nigeria. Programme and policy interventions should focus on enhancing socioeconomic status of women.

## Introduction

Adequate provision of maternal health care services such as antenatal care (ANC) and skilled birth assistance (SBA) is pivotal to achieving safe motherhood and enhanced child health outcomes. However, maternal health outcomes remain poor in sub-Saharan Africa (SSA). Maternal morbidity and mortality continue to be a serious public health concern despite appreciable decline across the world. While maternal mortality reduced globally by 38% between 2000 and 2017, sub-Saharan Africa experiences high maternal deaths. The region is responsible for two-thirds of maternal deaths globally in 2017 (1). An estimated 99% of all maternal deaths occur in developing countries and more than half occur in sub-Saharan Africa (2, 3). The situation of maternal health in Nigeria is worrisome and aligns with other countries in SSA. Maternal mortality ratio in Nigeria stands at 512 deaths per 100,000 live births with the country accounting for 10% of the global maternal mortality burden (4). The ratio increased from 545 to 575 deaths per 100,000 live births between 2008 and 2013 but reduced to 512 in 2018, reflecting a deplorable scenario and failure to achieve the 2015 Millenium Development Goal 5 (4). Low antenatal care attendance (ANC) and skilled delivery is associated with poor maternal health outcomes in Nigeria. The latest 2018 Nigeria Demographic and Health Survey showed that 57% of women had at least four ANC visits, deliveries by skilled birth attendants were 43%, immunization coverage for vaccination 21%, and modern contraceptive prevalence rate among currently married women was 12% (4).

A growing body of scholarly literature has examined a range of socioeconomic and demographic factors associated with antenatal care attendance and skilled delivery, reporting mixed results (5, 6, 7, 8). For instance, studies have found association between age, place of residence, level of education, wealth status and maternal health care services uptake (7–10). Most studies either linked IPV or male involvement separately to maternal healthcare uptake. The relationship between IPV and maternal health care uptake is conflicting. For instance, women who experienced IPV had lower odds of maternal health care uptake (11–13). However, other studies found no association between IPV and maternal health care uptake (13, 14). A study in the northern part of Nigeria reported ANC and skilled delivery (46.4%) and (22.1%), respectively (15). The study also found different prevalence rates of the three forms of intimate partner violence, including physical violence (16.7%), emotional violence (35.8%), and sexual violence (8.2%). The study further established that women who experienced IPV had lower odds of maternal health care uptake, with non-significant association after controlling for socio-demographic factors. Contrary to expectation, a study in Nigeria (16) found that women who were exposed to intimate partner violence had higher odds of using modern contraceptives. Another study found higher odds of non-use of modern contraceptives among women not exposed to intimate partner violence (14).

While the influence of male involvement on maternal health care uptake has been sparingly examined with some evidence of increasing uptake of ANC and skilled delivery (17, 18) in Nigeria, what is yet to be examined, however, is the mediating influence of intimate partner violence on male involvement and maternal health care services uptake. There is much evidence in the literature which associates IPV with adverse socioeconomic and health consequences in Nigeria. As a departure from previous study, we argue that intimate partner violence may truncate the positive influence of men's involvement in pregnancy-related care. In Nigeria, IPV remains very high as women who witness it during pregnancy have elevated risk of adverse maternal health outcomes, including pregnancy complications, stillbirths, induced abortion, injuries, and other depressive symptoms (15, 17). Meanwhile, the influence of male involvement on pregnancy care has been gaining traction in Nigeria despite deep-rooted patriarchal orientations which affect women's agency to utilise health care owing to their subservient position, as well as robust adherence of men to traditional gender norms which ostensibly label men who participate in pregnancy care as a weakling or a social deviant of masculinity orientation (17, 19). This may largely be responsible for low male involvement in pregnancy and other maternal health care services in Nigeria.

Male involvement in the context of abusive marital relationship is complicated. We argue that women are likely to be discouraged to seek maternal health care services owing to threat of recurrent marital abuse from their partners and its resultant grave consequences. Besides, the patriarchal context in Nigeria promotes intimate partner violence and this may limit male involvement in pregnancy care owing to gender power imbalance, traditional roles and societal expectations which make it challenging for men to actively participate in maternal health care, or for women to seek their partners' involvement (20). There is a need to understand the mediating influence of IPV on male involvement and maternal health care uptake for a number of reasons. One, this information is what policy makers, government and programmers need to design interventions which will enhance male involvement and maternal health outcomes. The findings from this study will contribute to knowledge base, inform policy makers and government at various levels to promote interventions aimed at addressing poor socioeconomic status and gender power inequalities in Nigeria. This study therefore assessed the separate and joint influence of IPV and male involvement in household economic and health decision-making on maternal health care uptake among women of reproductive age in Nigeria.

### Theoretical framework

Drawing on gender and power theory based on the exposition that gender-inequalities exist at hierarchical levels, which resonate with structural subordination of women in access to resources, power and their overall well-being. The subordinate position of women makes them unable to exert their agency in making health decisions which may be exacerbated by intimate partner violence. This theory echoes male dominance and subservient position of women as men needlessly or arbitrarily usurp women's decision-making autonomy in the scheme of things, including decision-making on their health. These gender inequalities are explained by three themes, namely sexual division of labour, sexual division of power and structure of cathexis (21). Unequal power relationships between men and women are couched in sexual division of power. Here, men dominate women in household decision-making owing to their subordinate position which is well-entrenched in African social milieu. The structure of cathexis is used in this study to lend credence to the restrictive sociocultural norms which affect women's belief system, propagate subordination to the dictates and arbitrary usurpation of sexual and reproductive rights of women by the male gender. This has implications for women's utilisation of maternal health care services as they are expected to seek the approval of partners and subject themselves to the whims and caprice of men as regards their decision on maternal health care uptake (21).

This notion resonates with the supposition that women in abusive relationship may not have the agency to make decision with regard to seeking healthcare services. Similarly, women exposed to intimate partner violence are likely to be subservient in their marital relationship, and as such, men in such patriarchal setting may oppose their utilisation of modern health facility care as a result of the male gender of the healthcare providers. Previous studies established that men resisted visitation of health facility by their partners owing to the fear of being attended to by male health providers (5, 19). There is some significant evidence in scholarly literature, especially among the Islamic faithful that men disapproved of visitation of modern health facilities by their partners as a result of some deep-rooted traditional and gender norms (20).

## Materials and methods

### Data source and study design

We conducted a cross-sectional analysis of data from the most recent Demographic and Health Survey in Nigeria. This study used a nationally representative secondary data and a quantitative approach. The 2018 Nigeria Demographic and Health Survey (NDHS) individual women recode was employed in this study. This survey is the most recent and part of the Demographic and Health Surveys (DHS) programme which the Inner-City Fund (ICF) provided technical assistance targeting women aged 15–49 years who were randomly selected from households across Nigeria. In this study, the DHS weighting factor was applied to derive 7,847 eligible women which were considered for analysis. The survey is a nationally representative data collected by the DHS programme on domestic violence, especially IPV, male involvement and maternal healthcare services (MHCS) utilisation, including antenatal care attendance and skilled birth assistance from women aged 15–49 years. Comprehensive information on research methodology and procedures of collection of data for the surveys can be found in the final reports (4). This cross-sectional sample survey design was considered as appropriate to examine relationship between intimate partner violence, male involvement in household decision-making and maternal health care uptake owing to the availability of data, timeliness and representative of the sample from the population. This research approach was to generalise from the sample to the population so that inference can be made about the influence of IPV and male involvement on maternal health care uptake.

### Variable measurement

#### Explanatory variables

The key explanatory variables in the study were intimate partner violence and male involvement in household economic and health decision-making. IPV was measured by physical, sexual and emotional violence. In the 2018 NDHS, data on intimate partner violence were created by asking women to respond to the question of having experienced any of the forms of IPV. The following responses were obtained, namely “never”, “often”, “sometimes” and “yes”. However, “not in the last month” were categorized into “no” for those who never experienced while “yes” for others. This variable was recorded in the domestic violence module of the 2018 NDHS. We derived IPV using the three typologies captured in the domestic violence modules of the survey. A woman is said to have experienced physical violence if there was a yes response to any of the questions, ditto sexual and emotional violence. More clearly, intimate partner violence was defined as having experienced any form of violence (physical, emotional, or sexual violence from intimate partners and categorized as “0” (i.e., experienced no form of violence) and “1” (i.e., had experienced any form of violence).

Physical violence was measured based on responses to being pushed by a partner, shook or threw something at you; slapped; twisted your arm or pulled your hair; punched you with his fist or with something that could hurt you; kicked, dragged or beat you up; tried to choke you or burn you on purpose; threatened or attacked you with a knife, gun, or any other weapon. Sexual violence was also measured by being physically forced by a partner, especially with threats, to have sexual intercourse with him even when you did not want to. Emotional violence was measured if partner said or did something to humiliate you in front of others, threatened to hurt or harm you, insulted you or made you feel bad about yourself.

The other principal explanatory variable was male involvement in household decision-making with respect to who makes decisions on healthcare and household expenditures. Information on male involvement was selected based on survey information that involved spousal decision-making in matters that pertain to the respondents. Issues such as decision on how spouse earning was spent, decision-making on use of contraceptives and decision on how a spouse's healthcare was made. These questions have the options “respondents alone” “respondents and husbands” and “husbands/partners alone”. Responses with “respondents and husbands” and “husbands/partners alone” were categorized as “male involved” while those with “respondents alone” were tagged “no male involvement”. Other control variables included age of women categorized as 15–24 years and 35 years and above. Education was also classified as none, primary, and secondary and tertiary combined. Place of residence was categorized into rural and urban area; wealth index was also categorized as used in the 2018 NDHS. Religion was categorized into Christianity, Islam and Traditional/others. Family type was categorized as monogamy and polygamy, and family size categorized as less than 5 children and 6 or more children.

#### Outcome variable

The outcome variables—antenatal care visit and skilled birth attendance were selected as the main measures of maternal healthcare services. Previous studies have considered ANC and skilled birth attendance as important measures of maternal healthcare services (11, 13, 22).

### Statistical analysis

Data were analysed using univariate, bivariate and multivariable analytical methods. At univariate level, we used charts, frequency and percentage distributions to determine the prevalence of antenatal care attendance, skilled delivery, male involvement and intimate partner violence. At bivariate level, we used Chi-square statistical test of independence to examine association between IPV, male involvement and maternal healthcare services utilisation, while at multivariable level, binary logistic regression model was used. The datasets were checked for missing values which were excluded and weighted with the suitable sampling weights designed in Nigeria Demographic and Health Survey (DHS) sampling technique using Stata software (version 14.1).

More specifically, this study adopted appropriate descriptive (frequency distribution and charts) and inferential statistics (Chi-square test and binary logistic regression). At the univariate level, descriptive statistics related to the characteristics of the study population, forms of IPV, male involvement and maternal healthcare service use (place of delivery and use of ANC) were generated through frequency and percentage. At bivariate level, the Pearson Chi-square test was used to test for statistical association between the variables. At multivariate level, three models were developed. Model 1 included relationship between IPV (emotional, physical and sexual violence) and maternal healthcare service use. Model 2 included IPV (emotional, physical and sexual violence), male involvement (decision-making on earnings and decision-making on healthcare) and maternal healthcare service use. Model 3 included IPV (emotional, physical and sexual violence), male involvement (decision-making on earnings and decision-making on healthcare), other control variables (age, place of residence, wealth status, level of education, religion, family size and family type) and maternal healthcare service use. However, decision making on contraceptive use was not included in the bivariate analysis and Model 2 and Model 3 of multivariable analysis owing to iteration and multicollinearity experienced with some variables.

### Ethical considerations

We used publicly available data in this study. The ethical procedures for data collection were the responsibility of the institutions that commissioned, funded, or managed the survey. All DHS is approved by ICF International and Institutional Review Board (IRB) to ensure that the protocols are in compliance with the U. S. Department of Health and Human Services regulations for the protection of human subjects. Thus, this study did not require further ethical approval. However, the authors were given approval to use the data.

## Results

### Socio-demographic characteristics of the respondents

Table 1 shows the result of selected socio-demographic characteristics of the respondents. Nearly two-fifths (41%) were below 30 years and 57% resided in rural areas. The proportion of women who had no education and those who had secondary or higher education were similar (41.7% vs. 42.2%), respectively. Most of the respondents belong to the richest wealth status, more than half 56% were Islamic faithful while 70% of the women were from monogamous families and 52% had a family size of 6 children or more.

**Table 1:** Percentage distribution of socio-demographic characteristics of the respondents.

Characteristics	Frequency	Percent
Age group		
Below 30	3,188	40.6
30–39	2,979	38.0
40–49	1,679	21.4
Place of residence		
Urban	3,378	43.1
Rural	4,469	57.0
Level of education		
None	3,274	41.7
Primary	1,263	16.1
Secondary/tertiary	3,310	42.2
Wealth status		
Poor	3,055	38.9
Medium	1,587	20.2
Richest	3,205	40.8
Religion		
Christian	3,388	43.2
Islam	4,416	56.3
Traditionalist/others	43	0.5
Family type		
Monogamy	5,531	70.5
Polygamy	2,316	29.5
Family size		
Less than 5	3,738	47.6
6 and more	4,108	52.4

### Male involvement in household and health decision-making

**Figure 1:**
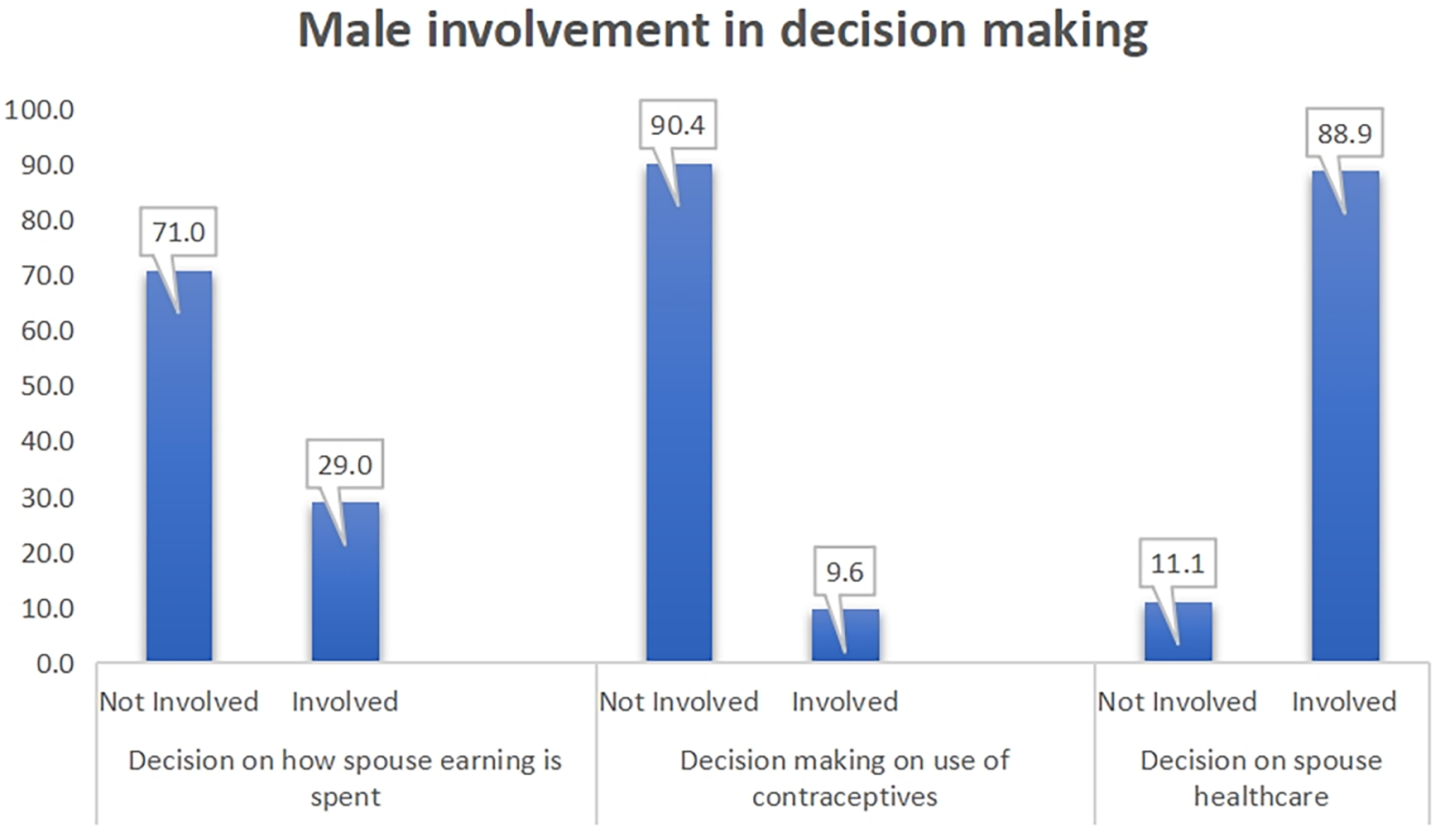
Information on male involvement in decision making.

This information on male involvement in decision making was based on the reports of women. The results showed that one-third of men were involved in decision on how spouse earnings were spent, 10% of men were involved in decision on women's use of contraceptive, while 89% reported male involvement in their healthcare (Figure 1).

### Experience of intimate partner violence

**Figure 2:**
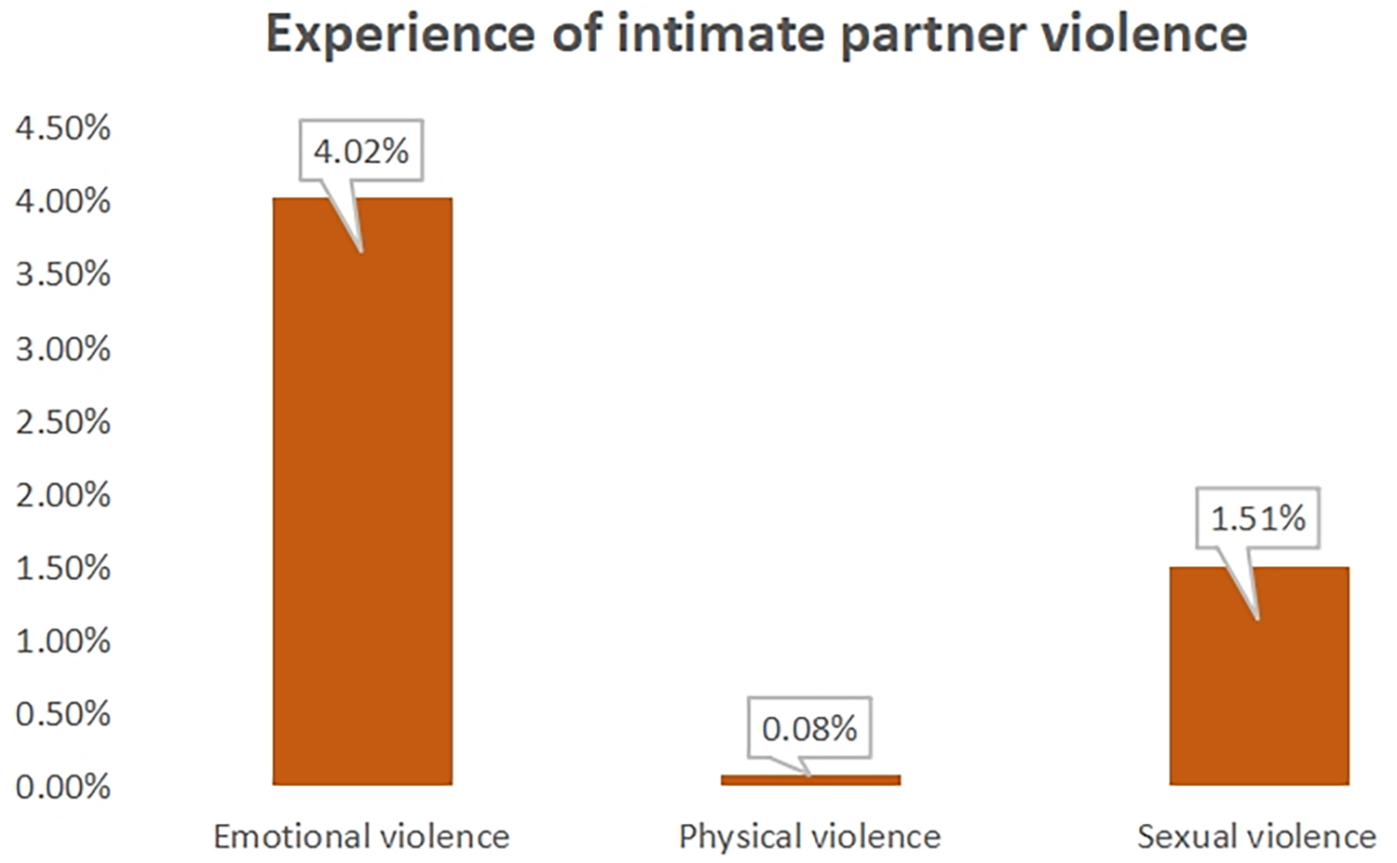
Experience of intimate partner violence among.

The vast majority of the respondents experienced emotional violence (4%) while (1.5%) witnessed sexual violence (Figure 2).

### Maternal healthcare services utilisation

**Figure 3:**
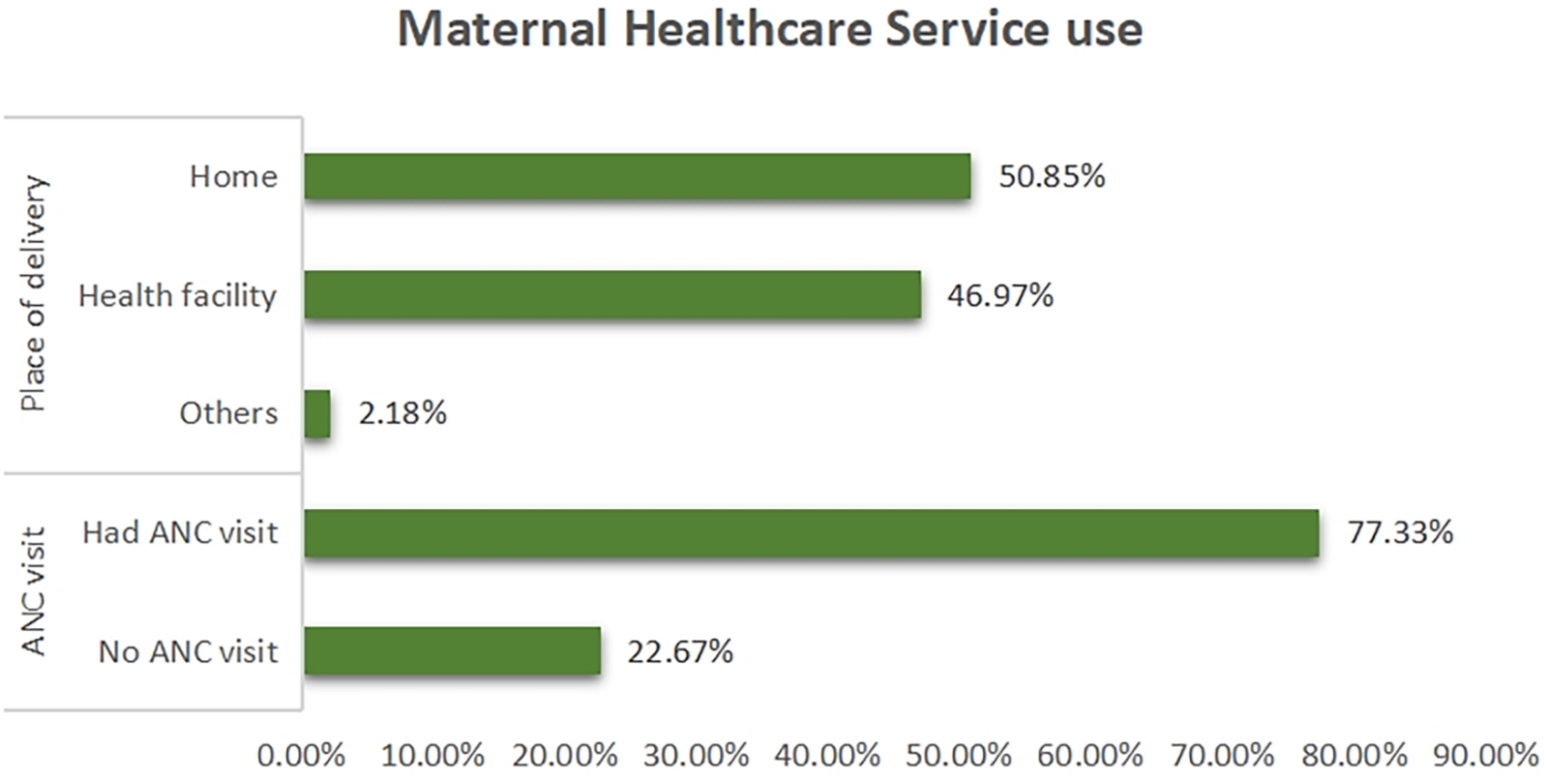
Maternal healthcare services utilisation.

The result revealed that 77% of the respondents visited the health facility for antenatal care. Also, almost half of the respondents (47%) used health facility for delivery while more than half delivered at home (Figure 3).

### Bivariate analysis

In Table 2, bivariate relationship between socio-demographic characteristics, intimate partner violence, male involvement, antenatal care attendance and skilled delivery was presented. Across the age groups, more than two-thirds of the women had ANC visit, with women 30–39 years having highest percentage (79%). However, more than half of the respondents had non-facility delivery. Similarly, maternal age showed significant association with ANC visit and place of delivery. Place of residence was also significantly associated with ANC. The results indicated urban women (89%) had ANC visit, and similarly, 66% of this group had facility delivery. The results showed 71% of rural women had non-facility delivery, indicating a high proportion of delivery outside modern health facilities in the countryside.

Additionally, wealth index, educational status, religion, family type and family size had significant association with ANC visit and place of delivery. Yet, more than 50% of the poor and the medium wealth status had ANC visit. This is similar for women with no education and primary education. While the vast majority across the religious groups had ANC visit, more than 50% of the women from Islam, traditional/other religion had non-facility delivery. Concerning intimate partner violence, physical, emotional and sexual violence had no association with ANC visit and place of delivery, though same proportion of women who experienced physical violence had ANC visit and facility delivery. However, three-quarters (75%) of women who experienced emotional violence had ANC visit and 55% had non-facility delivery. This is similar for women who experienced sexual violence.

While information on who decides on how to spend respondent's earnings was significant with place of delivery, it was not significant with ANC visit. Nevertheless, more than 60% of the respondents whose partners were involved in household decision-making had ANC visit, as well as facility delivery. Also, information on who decides on respondents' healthcare had significant association with ANC visit and place of delivery, with more than three-quarters of the respondents whose partners were involved in household and health decision-making had ANC visit, while more than half (57%) had non-facility delivery.

### Multivariable analysis: influence of intimate partner violence and male involvement on maternal healthcare services utilisation, controlling for socio-demographic factors

Table 3 shows multivariable analysis of the unadjusted relationship between intimate partner violence and maternal health care uptake in model 1 and the mediating influence of IPV on male involvement and maternal health care uptake in model 2. In model 3, relationship between IPV, male involvement and maternal health care uptake was considered, controlling for socio-demographic characteristics. There was no significant association between IPV and maternal health care uptake in model 1. Women who were emotionally and physically abused were less likely to attend antenatal care (OR = 0.94; C.I: 0.66–1.32), (OR = 0.30; C.I: 0.03–2.64), respectively. However, women who were sexually abused were more likely to use antenatal care (OR = 1.13; C.I: 0.64–2.02). This is reasonable because sexual abuse could lead to unwanted pregnancy and as such necessitates antenatal care attendance. On the other hand, IPV was not significantly associated with skilled delivery. Women who were emotionally (OR = 0.85; C.I: 0.62–1.15), sexually abused (OR = 0.82; C.I: 0.50–1.35) were less likely to use skilled delivery. Nevertheless, women who were physically abused (OR = 1.80; C.I: 0.21–15.75) had higher odds of using skilled delivery.

**Table 3:** Multivariable analysis of influence of intimate partner violence and male involvement on maternal healthcare services utilisation, controlling for socio-demographic characteristics.

Characteristics	Antenatal care service			Place of delivery		
ANC use	Model 1	Model 2	Model 3	Model 1	Model 2	Model 3
Emotional violence						
No	1.0 (RC)	1.0 (RC)	1.0 (RC)		1.0 (RC)	1.0 (RC)
Yes	0.94 (0.66–1.32)	0.70 (0.44–1.10)	0.70 (0.42–1.18)	0.85 (0.62–1.15)	0.58 (0.39–0.85)**	0.49 (0.31–0.77)**
Sexual violence						
No		1.0 (RC)	1.0 (RC)		1.0 (RC)	1.0 (RC)
Yes	1.13 (0.64–2.02)	3.20 (1.20–8.50)*	3.38 (1.21–9.38)*	0.82 (0.50–1.35)	0.85 (0.43–1.66)	0.72 (0.29–1.79)
Physical violence						
No		1.0 (RC)	1.0 (RC)		1.0 (RC)	1.0 (RC)
Yes	0.30 (0.03–2.64)	0.10 (0.01–1.08)	0.02 (0.00–0.43)*	1.80 (0.21–15.73)	1.85 (0.25–13.84)	0.60 (0.05–7.54)
Person who makes decision on spending wife's earning						
No male involvement		1.0 (RC)	1.0 (RC)		1.0 (RC)	1.0 (RC)
Male involvement		1.19 (0.92–1.55)	0.72 (0.55–0.96)*		2.15 (1.79–2.56)*	1.20 (0.98–1.48)
Person who makes decision on wife/partner's health care						
No male involvement		1.0 (RC)	1.0 (RC)		1.0 (RC)	1.0 (RC)
Male involvement		0.64 (0.44–0.94)*	0.89 (0.57–1.39)		0.46 (0.34–0.62)***	0.66 (0.49–0.89)**
Age						
Below 30			1.0 (RC)			1.0 (RC)
30–39			1.02 (0.76–1.36))			1.38 (1.08–1.78)*
40–49			0.71 (0.48–1.03)			1.27 (0.91–1.78)
Place of residence						
Urban			1.0 (RC)			1.0 (RC)
Rural			0.58 (0.42–0.80)**			0.60 (0.49–0.75)***
Level of education						
None			1.0 (RC)			1.0 (RC)
Primary			2.20 (1.49–3.23)***			2.49 (1.79–3.47)***
Secondary/tertiary			3.64 (2.48–5.34)***			4.47 (3.32–6.03)***
Wealth status						
Poor			1.0 (RC)			1.0 (RC)
Medium			1.77 (1.27–2.48)**			1.69 (1.26–2.27)***
Richest			3.33 (2.35–4.72)***			3.00 (2.25–4.00)***
Religion						
Christian			1.0 (RC)			1.0 (RC)
Islam			1.29 (0.92–1.81)			0.57 (0.45–0.72)***
Traditionalist/others			0.60 (0.15–2.48)			0.94 (0.21–4.28)
Family type						
Monogamy			1.0 (RC)			1.0 (RC)
Polygamy			0.80 (0.60–1.07)			0.72 (0.55–0.95)*
Family size						
1–5 members			1.0 (RC)			1.0 (RC)
6 and more			1.02 (0.80–1.30)			0.80 (0.65–0.99)*

RC, reference category.

* *p* < 0.05.

** *p* < 0.01.

*** *p* < 0.001.

**Table 2:** Association between socio-demographic characteristics, intimate partner violence, male involvement and maternal healthcare services utilisation.

Characteristics	ANC visit			Place of delivery		
	No ANC visit	Had ANC visit		Non facility delivery	Health facility delivery	
	*N* (%)	*N* (%)	Statistics	*N* (%)	*N* (%)	Statistics
Age						
Below 30	675 (24.5)	2,074 (75.5)	χ^2^ = 37.626	1,605 (58.4)	1,144 (41.6)	*χ*^2^ = 40.408
30–39	504 (20.7)	1,934 (79.3)	*p* < 0.001	1,237 (50.8)	1,200 (49.2)	*p* < 0.001
40–49	209 (31.2)	460.7 (68.8)		403 (60.1)	267 (39.9)	
Place of residence						
Urban	275 (10.8)	2,274 (89.2)	*χ*^2^ = 451.973	876 (34.4)	1,673 (65.6)	*χ*^2^ = 879.202
Rural	1,113 (33.6)	2,195 (66.4)	*p* < 0.001	2,369 (71.6)	939 (28.4)	*p* < 0.001
Wealth index						
Poor	946 (41.2)	1,352 (58. 8)		1,893 (82.4)	405 (17.6)	
Medium	246 (20.4)	961 (79.6)	*χ*^2^ = 762.816	696.8 (57.7)	510 (42.3)	*χ*^2^ = 1,522.744
Richest	196 (8.34)	2,156 (91.7)	*p* < 0.001	655 (27.9)	1,696 (72.1)	*p* < 0.001
Level of education						
None	1,026 (42.2)	1,402 (57.8)		2,061 (84.9)	367 (15.1)	
Primary	151.8 (17.4)	722.6 (82.6)	*χ*^2^ = 890.464	490 (56.0)	384 (44.0)	*χ*^2^ = 1,822.933
Secondary/tertiary	210.1 (8.2)	2,344 (91.8)	*p* < 0.001	694 (27.2)	1,860 (72.8)	*p* < 0.001
Religion						
Christianity	304.8 (12.5)	2,134 (87.5)		761 (31.2)	1,678 (68.8)	
Islam	1,074 (31.7)	2,316 (68.3)	*χ*^2^ = 314.748	2,467 (72.8)	924 (27.2)	*χ*^2^ = 1,077.175
Traditionalist/others	8.642 (31.6)	18.68 (68.4)	*p* < 0.001	76 (69.7)	10 (38.3)	*p* < 0.001
Family size						
1–5	497.1 (18.8)	2,143 (81.2)	*χ*^2^ = 68.320	1,190 (45.1)	1,450 (54.9)	*χ*^2^ = 255.049
6 and more	890.5 (27.7)	2,326 (72.3)	*p* < 0.000	2,055 (63.9)	1,162 (36.1)	*p* < 0.000
Family type						
Monogamy	820.6 (19.4)	3,402 (80.6)	*χ*^2^ = 165.053	2,002 (47.4)	2,221 (52.6)	*χ*^2^ = 425.230
Polygamy	566.9 (34.7)	1,067 (65.3)	*p* < 0.000	1,243 (76.1)	391 (23.93)	*p* < 0.000
Physical violence						
No	1,386 (23.7)	4,283 (76.4)	*χ*^2^ = 1.534	3,243 (55.4)	2,610 (44.6)	*χ*^2^ = 0.055
Yes	2 (50)	2 (50)	*p* = 0.253	2 (50.0)	2 (50.0)	*p* = 0.829
Emotional violence						
No	1,326 (23.6)	4,283 (76.4)	*χ*^2^ = 0.236	3,097 (55.2)	2,512 (44.8)	*χ*^2^ = 2.145
Yes	62 (24.9)	186 (75.1)	*p* = 0.680	148 (59.6)	100 (40.25)	*p* = 0.221
Sexual violence						
No	1,365 (23.7)	4,395 (76.3)	*χ*^2^ = 0.032	3,186 (55.3)	2,574 (44.7)	*χ*^2^ = 1.337
Yes	22 (23.0)	74 (77.0)	*p* = 0.88	71 (58.9)	50 (41.1)	*p* = 0.336
Decision on person who decides how to spend respondent's/wife's earnings						
No male involvement	477 (18.2)	2,144 (81.8)	*χ*^2^ = 1.986	1,452 (55.4)	1,169 (44.6)	*χ*^2^ = 90.96
Male involvement	171 (16.3)	883 (83.7)	*p* = 0.303	407 (38.6)	648 (61.4)	*p* < 0.001
Decision on who makes decision on respondent's/wife's health care						
No male involvement	108 (17.9)	494 (82.1)	*χ*^2^ = 13.38	253 (40.3)	359 (59.7)	*χ*^2^ = 67.56
Male involvement	1,280 (24.3)	3,975 (75.7)	*p* < 0.013	3,003 (57.1)	2,252 (42.9)	*p* < 0.001

In model 2, the mediating influence of IPV on male involvement resulted in women who experienced sexual violence more likely to have antenatal care attendance (OR = 3.20; C.I: 1.20–8.50). Additionally, women whose partners were involved in health decision making had lower odds of antenatal care attendance (OR = 0.64; C.I: 0.44–0.94). This reflects that domination of women by their partners in relation to how to spend their earnings may limit availability of medical costs and thus reduces antenatal care attendance. On the other hand, the mediating influence of IPV on male involvement, in model 2, resulted in a significant association and lower likelihood for women who were emotionally abused to use skilled delivery (OR = 0.58; C.I: 0.39–0.85). In addition, women whose partners were involved in the spending of their earnings had higher odds of using skilled delivery (OR = 2.15; C.I: 1.79–2.56). Yet, women whose partners were involved in their health decision-making had lower odds of using skilled delivery (OR = 0.46; C.I: 0.34–0.62).

In model 3, the influence of IPV and male involvement on use of antenatal care, controlling for socio-demographic factors indicated that women who experienced sexual violence had higher likelihood of antenatal care attendance (OR = 3.38; C.I: 1.21–9.38) but women who witnessed physical violence had lower odds of antenatal care attendance (OR = 0.02; C.I: 0.00–0.43). Women whose partners were involved in the spending of their earnings had lower probability of antenatal care attendance (OR = 0.72; C.I: 0.55–0.96). Women who reside in the rural areas were less likely to have antenatal care attendance (OR = 0.58; C.I: 0.42–0.80) and those who had primary education (OR = 2.20; C.I: 1.49–3.23) and secondary/tertiary education (OR = 3.64; C.I:2.48–5.34) were more likely to have antenatal care attendance. Results indicated that women in the medium (OR = 1.77; C.I: 1.27–2.48) and the richest (OR = 3.33; C.I: 2.35–4.72) categories were more likely to have antenatal care attendance. In model 3 on the other hand, the mediating influence of IPV on male involvement and other control variables reflected in the odds of skilled delivery. The results indicated that women exposed to emotional violence were less likely to use skilled delivery (OR = 0.49; C.I: 0.31–0.77). Women whose partners were involved in their health decision making had lower likelihood of using skilled delivery (OR = 0.66; C.I: 0.49–0.89). Further, the result showed that women aged 30–39 years (OR = 1.38; C.I: 1.08–1.78), primary education (OR = 2.49; C.I: 1.79–3.47), secondary/tertiary education (OR = 4.47; C.I: 3.32–6.03), medium wealth category (OR = 1.69; C.I: 1.26–2.27) and richest wealth category (OR = 3.00; C.I: 2.25–4.00) had higher likelihood of using skilled delivery. However, rural dweller (OR = 0.60; C.I: 0.49–0.75), Islamic religion (OR = 0.57; C.I: 0.45–0.72), polygamous family type (OR = 0.72; C.I: 0.55–0.95), family size of 6 or more (OR = 0.80; C.I: 0.65–0.99) had lower odds of using skilled delivery.

## Discussion

This study examined the influence of intimate partner violence and male involvement on maternal health care services uptake in Nigeria. The study employed a nationally representative secondary data which collected information on domestic violence, women's decision-making autonomy and maternal health care services utilisation and revealed some salient findings. Intimate partner violence is a serious public health challenge which has grave psycho-social, economic and physical health consequences. We found the proportions of women who experienced sexual (1.5%), emotional (4.0%) and physical violence (0.08%) in this study. This is contrary to previous studies conducted in Nigeria which reported a larger proportion of women who experienced IPV (7, 16). For instance, Solanke (13) reported a higher prevalence of IPV, as well as a study by Adewoyin et al. (15) which found 41% physical violence, 38% sexual violence and 29% emotional violence among women in Northern Nigeria. The plausible explanation for the divergence could be different settings of the studies and measures used to compute intimate partner violence. The policy implication is that regardless of the smaller magnitude of IPV reported in this study, efforts should be made to nip it in the bud owing to its adverse health and social consequences.

This study also reported 49.9% of skilled health delivery and 77% antenatal care visits. These results are in agreement with previous studies which reported very similar statistics (6, 10, 23). There are plausible explanations for the convergence of results and why antenatal care was higher than skilled delivery. One of the probable reasons is that some women who attended antenatal care might have their deliveries outside health facilities. A number of studies reported higher antenatal care attendance than skilled health delivery in Nigeria (10, 13, 22). This lends credence to the fact that more than half of women deliver in non-facility health in Nigeria. The 2018 Nigeria Demographic and Health Survey and a Nigerian study reported similar prevalence rates of antenatal care visits and skilled delivery (4, 15). This study also reported that 88.9% of women reported that their partners were involved in pregnancy related care. There is a need to strengthen policies which encourage male involvement in pregnancy care. Our result is not in agreement with previous studies which reported that more than 30% of women lacked involvement in matters relating to their reproductive health (24, 25) and another similar study indicated more than 40% of women could not resist sexual intercourse from their partners and 50% could not request their partners to use condom (26). The probable reason for the divergence of result is the different proxy variables used in measuring male involvement across studies. This echoes women's lack of ability to make independent decision with regard to their sexual matters.

At the bivariate level, this study found significant relationship between age of women, level of education, family size, family type, place of residence and wealth index and use of antenatal care and skilled delivery. These results have implications for uptake of maternal health care services because women with improved socioeconomic status are better placed to using health facilities for antenatal care and skilled delivery. For instance, women with higher educational level, urban resident, monogamy and moderate family size had higher proportion of maternal health uptake. Similar findings have been reported in prior studies (3, 6). There is a need to focus intervention on enhancing women's socioeconomic status. However, IPV was not significantly associated with maternal health care uptake. Mixed findings have been reported in previous studies (15, 22). Plausible explanation for this finding could be the small proportion of women exposed to IPV in this study. This negates our hypothesis which states that there is significant relationship between IPV and maternal health care uptake even with conflicting evidence in scholarly literature. Yet, male involvement was significantly associated with maternal health care services uptake. This result lends credence to prior studies which established that male involvement in decision-making influences maternal health uptake (17, 18, 27). Many studies have established that male involvement in reproductive health care services is vital for positive health outcomes. For example, men's participation in pregnancy care services enhanced the reproductive health information and couple's concordance on pregnancy care instructions (17). A study in Vietnam also indicated that men's participation in a programme which was focused on promoting exclusive breastfeeding and counselling among couples increased exclusive breastfeeding, enhanced positive attitudes and adequate knowledge (28).

At the multivariable level, our results revealed that IPV was not significantly associated with maternal health care uptake in model 1. This result echoes previous inconsistent patterns of relationships between IPV and maternal health care services utilisation. A study revealed that IPV has a significant relationship with modern contraceptive use (16). Prior studies have shown inconsistent relationship between IPV and maternal health care services uptake (15, 22). Plausible reason could be that women who experienced intimate partner violence decide to have skilled delivery in a context of health challenge or during emergency, even without partners' consent. The non-significance could also be as a result of the small proportion of frequencies of the three typologies of IPV in this study. This speaks to the need to provide health facility delivery for women who are domestically abused. In model 2, women who were sexually abused had higher odds of antenatal care visit while women who were emotionally abused were less likely to have skilled delivery. The inconsistent patterns of findings on the influence of IPV on maternal health care services utilisation have been reported in many studies (11, 12). Plausible reasons for the inconsistent findings are many. One possible reason is the setting in which the abuse occurred and another is the different patterns of IPV. Another reason is the mediating influence of IPV on male involvement. The results showed that the influence of male involvement may be truncated because IPV may lead to lower antenatal care attendance. This result suggests that women who are exposed to IPV even in the context of male participation in reproductive and economic decisions may have reduced antenatal care attendance. The reason for higher use of skilled delivery for women who experienced physical abuse may be because of emergency health needs. The implication is that male involvement in pregnancy care may not reduce exposure of women to IPV. Pregnant women who are physically abused may seek skilled delivery care because of the precarious situation and debilitating impacts of physical harm.

Furthermore, this study revealed conflicting results for women whose partners were involved in their health expenditures decision-making with regard to antenatal care attendance and skilled delivery. These results echo the stance that male involvement enhances pregnancy-related care. The plausible reason is that women whose partners are involved in the spending of their earnings, even in an abusive relationship have a higher probability of using health facility for delivery but not antenatal care attendance. Skilled delivery for women in a context of abusive relationship and male participation may not be limited. However, this study showed a limiting influence on antenatal care attendance. Male involvement in health decision in an abusive relationship may affect antenatal care attendance because it resonates gender inequality and submissiveness of women to the dictates of men in relation to decision-making on their health. The threat of IPV may limit male involvement and antenatal care attendance.

This confirms our hypothesis that male involvement is associated with maternal health care uptake and validates the theory of gender and power which argues that social and cultural norms can promote undue submissiveness of women which result in gender-based power inequality and poor sexual and reproductive health outcomes. The policy implication is to strengthen intervention which promotes male involvement in pregnancy-related care even in a context of abusive relationship regardless of the seemingly difficult situation. Yet, women whose partners were involved in decision-making with regard to the spending of their earnings were more likely to use skilled delivery even in a context of IPV. This speaks to involvement of men in household decision-making and its impacts on skilled delivery.

In model 3, socio-demographic factors were controlled for. This study revealed inconsistent patterns of results. However, improved socioeconomic status of women indicated a higher uptake of maternal health care services. Several studies have established that women with improved socioeconomic status had higher uptake of antenatal and skilled delivery net of IPV and male involvement (5, 7). This confirms the gender and power theory which argues that gender inequality and sociocultural norms make the male gender dominate women in all facets of life. This resonated in the thesis of this work. The study found that women in the rural areas, large family size, Islamic religion and polygyny family structure were less likely to have antenatal care visit and skilled delivery. Prior studies have confirmed that poor socioeconomic status of women influenced poor maternal health care uptake (9, 10). We argue that rural dweller in hard-to-reach populations lack adequate health care resources. Large family size and polygamous settings may also expose women to unnecessary rivalry among wives and paucity of resources, limiting their access to maternal health care services (29). Scholarly literature has also shown the limiting influence of Islamic religion on use of maternal health care services owing to gender-based norms, such as the sex of the healthcare providers attending to a pregnant woman and restriction of movement to public space. These results have implication for improving women's socioeconomic status, especially the Islamic faithful.

### Strengths and limitations

This study has strengths and limitations. The study used a nationally representative data which allowed external validity or wider generalisation of results outside Nigeria. This study also considered concepts that have not been explored jointly as exposures, male involvement and IPV, to tease out their relationships on maternal health care services uptake. Yet, this study has a few limitations. Some proxy variables were used for male involvement owing to lack of adequate measurements and the secondary nature of the data set. Other important indicators of male involvement in pregnancy care would have been considered in a primary data. Also, the results of this study may be affected by the retrospective nature of the data as memory lapse may crept in. The cross-sectional nature of the study may also affect causality because of temporal effect. This study focuses on association between variables but not a cause-effect analysis. Despite these limitations, this study has contributed significantly to scholarly literature and shifted knowledge base being the first study which examined the mediating influence of intimate partner violence on male involvement and maternal health care uptake on a national scale in Nigeria.

## Conclusion

This study established that male involvement influenced maternal health care uptake even in the context of abusive sexual relationships. The study also confirmed that women's socioeconomic status influenced maternal health care uptake in the context of IPV and male involvement in household spending and health decision-making. This speaks volume on the need to encourage improved women's status and also strengthen male involvement to mitigate the inimical influence of IPV on maternal health care services uptake among women in Nigeria.

## Data Availability

The datasets presented in this study can be found in online repositories. The names of the repository/repositories and accession number(s) can be found below: www.measuredhs.org.
